# Quantitative and qualitative characterization of commercially available oral suspension of probiotic products containing *Bacillus Clausii* spores

**DOI:** 10.1186/s12866-022-02631-w

**Published:** 2022-09-17

**Authors:** Akash Kharwar, Mohd Rabi Bazaz, Manoj P. Dandekar

**Affiliations:** Department of Biological Sciences, Department of Pharmacology and Toxicology, National Institute of Pharmaceutical Education and Research (NIPER), Balanagar, Hyderabad, Telangana 500037 India

**Keywords:** *Bacillus clausii*, Probiotics, Diarrhoea, Spore count, Antibiotic resistance

## Abstract

Probiotics contain beneficial live bacteria that confer several health benefits to the host. For the past 50 years, spore-forming *Bacillus* species have been used in the form of probiotics. Among these, *Bacillus clausii* strains are used for the management of acute and antibiotic-associated diarrhoea. In the present work, we have evaluated the asserted label information on randomly chosen commercial *Bacillus clausii* spore suspension of probiotic products. The quality and number of viable bacteria were evaluated based on the colony count, antibiotic resistance, and hemolytic activity assays. The colony fingerprinting and 16S rRNA gene-sequencing techniques were used to confirm the presence of a univariate strain (*Bacillus clausii*). Our results corroborated the label count of 2 × 10^9^ CFU/5 mL in BACIPRO®, ENTEROGERMINA®, and TUFPRO® products. However, vegetative spore count was not found to match with the given label count in BENEGUT®, PROALANA-B®, β-LOCK®, and PROCILLUS® *Bacillus clausii* brands. In the hemolytic activity assay, except for β-LOCK®, the other 6 products showed gamma-hemolysis activity. *Bacillus clausii* isolated from all 7 probiotic products demonstrated resistance to several broad-spectrum antibiotics. The 16S rRNA gene-sequencing data detected genera of *Bacillus* and *Bacillus clausii* strain in the BACIPRO®, ENTEROGERMINA®, PROALANA-B®, BENEGUT®, and TUFPRO® products; however, *Ralstonia mannitolilytica* and *Paenibacillus dendritiformis* species were identified in β-LOCK**®** and PROCILLUS®, respectively. As correct label information was observed only in BACIPRO®, ENTEROGERMINA®, and TUFPRO® products, it is proposed that a more stringent quality check would minimize the possibility of mismatch concerning the label information.

**Supplementary Information:**

The online version contains supplementary material available at 10.1186/s12866-022-02631-w.

## Introduction

Probiotics contain vital non-pathogenic bacteria which transiently colonize the intestine and offer positive health benefits to the host by increasing the count of beneficial commensal microbes [1, 2]. Therapeutic applications of probiotics have been demonstrated in the management of diarrhea, respiratory infections, and gastrointestinal diseases. Most of the probiotic preparations contain *Lactobacillus* and *Bifidobacterium sps*, which have been proven to improve the intestinal microenvironment of the host [3] and treat acute diarrhea in children [4]. *Bacillus clausii* containing probiotics have been used for the treatment of intestinal infections through the promotion of cellular and humoral immune activity [5]. The *Bacillus* species are ubiquitously present in the healthy gut accounting for around 2 × 10^6^ endospores [6]. The therapeutic benefits of *Bacillus clausii* were demonstrated in 1960 for the treatment of viral diarrhea in children and antibiotic-generated gut disturbances [7]. *Bacillus clausii* spores are known to survive in the environment of gastric pH and colonize the intestinal tract mucosa, and grow into vegetative forms [7]. Moreover, this bacterium is resistant to most antibiotics due to the presence of antibiotics-resistance genes, and thus it easily colonizes the digestive tract even in the presence of antibiotics [8]. The bacterium is also resistant to certain anti-infective agents [9]. Consequently, easy colonization, invulnerability, incitement, and antimicrobial properties of *Bacillus clausii* strain render it a preferable probiotic option.

Probiotic formulations of *Bacillus clausii* have been reported to be clinically efficacious for the treatment of acute diarrhea in adults [7, 10] and are considered safe except for a few reported incidences of sepsis and bacteremia in recent research [11, 12]. Diarrhea is the third leading cause of mortality in children under the age of five, accounting for 13% of mortality in this age group, killing around 300,000 children and 525,000 people each year in India and under-developed countries [13, 14]. The clinical importance of *Bacillus clausii* has also been corroborated in children with acute diarrhea as an additional therapy to oral rehydration therapy [15, 16]. A range of spore-forming *Bacillus clausii* probiotics are readily available over-the-counter (OTC) for the treatment of acute diarrhea in adults and children and as adjunctive therapy for *Helicobacter pylori* infection in India [17]. However, several studies have raised concerns regarding the conformity of marketed probiotic preparations [18, 19]. In India, Nutrition policy and the Indian Council of Medical Research and Department of Biotechnology (ICMR-DBT) guidelines [20–22] have recommended verification of the viable bacterial count, microbial species, and strains as per the product label. Accordingly, stringent consideration is required for the assessment of quality and guidelines of the probiotics before launching the products in the market.

In India, several spore-forming *Bacillus clausii* probiotics are available OTC for human use such as ECOGRO**®**, ENTEROGERMINA**®**, ENTROMAX**®**, OSPOR**®**, GUTPRO**®**, CYFOLAC**®,** BACIPRO**®,** β-LOCK®, BENEGUT®, PROCILLUS®, PROALANA-B®, and TUFPRO**®** [22–24]. As the therapeutic efficacy of probiotics is attributed to the specific bacterial strains and number of viable bacteria, a disparity between the label information on probiotics has raised concerns about the conformity of marketed probiotic preparations. Thus, considering the importance of an unbiased research facility evaluation, this study aimed to examine the possible mismatches in the asserted label information in 7 commercially available *Bacillus clausii* spore suspension probiotic products, BACIPRO**®**, ENTEROGERMINA®, β-LOCK®, BENEGUT®, PROCILLUS®, PROALANA-B®, and TUFPRO®. These formulations were assessed for colony count (CFU/mL), antibiotic resistance, and hemolytic activity. The 16S rRNA gene sequencing was performed to verify the presence of a univariate strain (*Bacillus clausii*). Since probiotic-based therapies are in great demand in India and worldwide, it is hypothesized that the current study may be pertinent for a precise check on the quality and quantity of probiotic preparations.

## Materials and methods

### Oral suspension of probiotics containing *Bacillus clausii* spores

We collected 7 commercially available spore-forming oral suspension products of the *Bacillus clausii* strain marketed in India. These products are available with the following brand names: BACIPRO®, ENTEROGERMINA®, β-LOCK®, BENEGUT®, PROCILLUS®, PROALANA-B®, and TUFPRO®. The manufacture/supplier information and batch number of the selected products are presented in Table 1. To rule out the batch-specific variation, we randomly chose 3 different batches of each product. However, due to the unavailability of different batches, only one lot of β-LOCK®, BENEGUT®, PROCILLUS®, and PROALANA-B® products were selected. We obtained 10 vials of each brand and stored them at a temperature not exceeding 30 °C.

**Table 1:** Details of commercial *Bacillus clausii* spore suspension probiotics

SN	Product Name	Manufacturer/Supplier	Batch No.	Strain Name	Label dose
1.	BACIPRO**®**	Unique Biotech Pvt. Limited, India	B0421 B0621 B0521	*Bacillus clausii* UBBC- 07	2 × 10^9^ spores/ 5 mL
2.	**TUFPRO®**	Unique Biotech Pvt. Limited, India	ZEY009 ZEY008 ZEY007	*Bacillus clausii* UBBC- 07	2 × 10^9^ spores/ 5 mL
3.	ENTEROGERMINA**®**	Sanofi-Synthelabo India Pvt. Limited	OI197 OI107 OI131	Four antibiotic-resistant *Bacillus clausii* strains (SIN, O/C, T, N/R)	2 × 10^9^ spores/ 5 mL
4.	**β**-LOCK**®**	Genetek Lifesciences Private Limited, India	BC20001	Not Available	2 × 10^9^ spores/ 5 mL
5.	BENEGET**®**	Virchow Biotech Private Limited, India	VBF0134	Not Available	2 × 10^9^ spores/ 5 mL
6.	PROALANA-B**®**	Virchow Biotech Private Limited, India	CBO4620	Not Available	2 × 10^9^ spores/ 5 mL
7.	PROCILLUS**®**	Virchow Biotech Private Limited, India	CTO1720	Not Available	2 × 10^9^ spores/ 5 mL

### Viable spore count and isolation of bacteria

The isolation and enumeration of bacteria were performed by the pour-plate method as described earlier [22]. In brief, isolation, and cultivation of bacteria were done in brain heart infusion (BHI) media and BHI agar. Typically, 5 mL of oral suspension was diluted with an equal volume of saline and vortexed. This stock solution was then serially diluted to obtain 10^6^, 10^7^, and 10^8^ dilutions of each product. Before plating, spores of the different samples were heat-killed at 75 °C for 25 min, to ensure the absence of any residual vegetative cells or germinated spores. Next, spores were plated and allowed to incubate at 44 °C for 48 h. Post-incubation visible colonies were counted and expressed as CFU.

The spread plate procedure was performed as described previously [25]. The same dilutions made for the pour-plate method were used for this procedure. Briefly, 100 μL of the sample was transferred aseptically to BHI 2% agar plate, and then the sample was uniformly spread using a Z glass rod. The plates were incubated at 37 °C for 24 h. The bacterial counting was manually done by two independent researchers using the microbiological plating method.

### Molecular characterization: Colony fingerprinting PCR

**Fig. 1:**
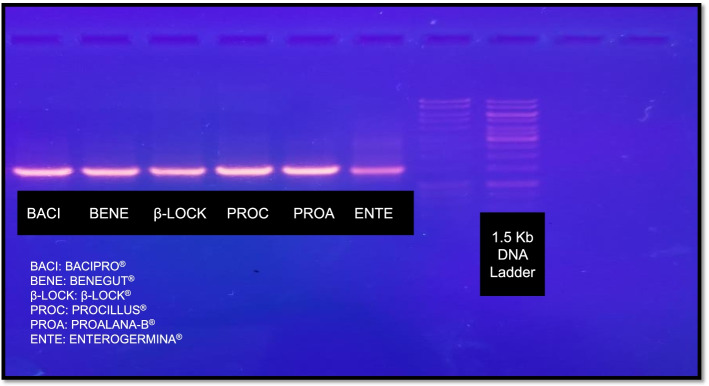
SDS-PAGE of the genomic DNA (gDNA): the agarose gel electrophoresis image represents the integrity of the isolated gDNA

**Fig. 2:**
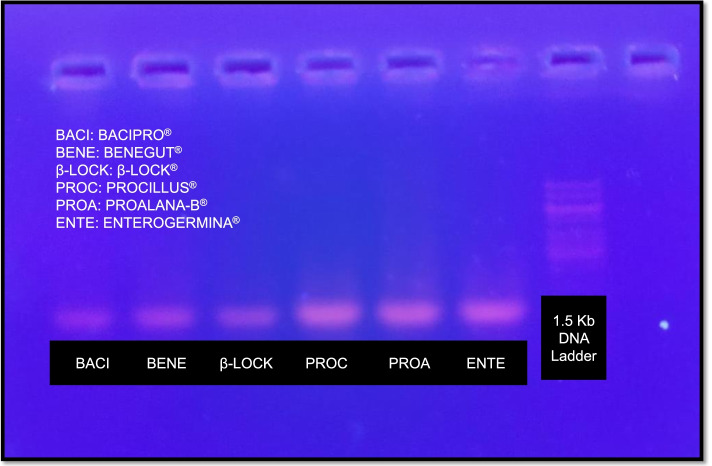
PCR gel: the agarose gel electrophoresis image indicates the integrity of amplified PCR products

The bacterial colony was isolated using the streak plate method. The bacterial DNA was extracted by phenol:chloroform:isomyl method as reported earlier [26] and DNA purity and integrity was confirmed by agarose gel electrophoresis (Fig. 1). DNA templates were amplified using 2 μL of forward and reverse primers, 20 μL of master mix (Taq polymerase, 10x buffer, Mg^2+^ ions, disH_2_O, Green Taq color), and RNASE free water to achieve the final reaction mixture of 40 μL. A thermal cycler was used to perform PCR amplification, which included an initial denaturation phase (95 °C for 7 min), 30 cycles of denaturation (90 °C for 30 s), annealing (40 °C for 1 min), extension (65 °C for 8 min), and a single final extension step (65 °C for 16 min). The PCR products were electrophoresed in 8% (w/v) agarose gel and the resulting fingerprints were compared directly with 1.5 kb DNA ladder under UV transilluminator after staining with ethidium bromide (Fig. 2).

### Antibiotic susceptibility of probiotic products

The disc diffusion methodology was used to perform the antimicrobial susceptibility test as reported earlier [27]. Thirty-one commercially available paper antibiotic discs with a defined concentration were employed. The results were categorized as susceptible when zone of inhibition diameter was 10 mm or more, and no inhibition zone diameter was considered as resistant [28]. The composition of the employed antibiotics is mentioned in Table 2.

**Table 2:** Composition of antibiotics

SN	Antibiotic name	Abbreviations	Disc concentration
1	Clarithromycin	CLR	15 mcg
2	Cefazolin	CZ	30 mcg
3	Cefoperazone	CPZ	75 mcg
4	Cefixime	CFM	5 mcg
5	Chloramphenicol	C	10 mcg
6	Streptomycin	S	25 mcg
7	Fluconazole	FLC	10 mcg
8	Metronidazole	MT	5 mcg
9	Novobiocin	NV	30 mcg
10	Ciprofloxacin	CIP	10 mcg
11	Erythromycin	E	5 mcg
12	Erythromycin	E	15 mcg
13	Amoxicillin	AMX	25 mcg
14	Rifampicin	RIF	5 mcg
15	Ofloxacin	OF	5 mcg
16	Penicillin-G	P	10 units
17	Azithromycin	AZM	15 mcg
18	Neomycin	N	30 mcg
19	Kanamycin	K	5 mcg
20	Streptomycin	S	10 mcg
21	Cefdinir	CDR	5 mcg
22	Straconazole	IT	10 mcg
23	Tetracycline	TE	10 mcg
24	Amoxiclav	AMC	30 mcg
25	Kanamycin	K	30 mcg
26	Fusidic acid	FC	30 mcg
27	Amikacin	AK	30 mcg
28	Nystatin	NS	100 units
29	Nalidixic acid	NA	30 mcg
30	Gentamycin	GEN	50 mcg
31	Clindamycin	C	10 mcg

The enriched BHI broth was prepared and one colony from the previous streaked plate of a sample was inoculated in the broth. This broth was incubated at 37 °C ± 2 °C for 24 h. In the culture inoculated agar plate, 31 commercially manufactured paper antibiotic discs of various doses were placed. The diameter of the zone (mm) indicates the measure of the susceptibility of the isolate and the amount of drug diffused through the agar medium. Each batch of individual products was tested independently using microbiological plating and molecular techniques to determine whether the strain was resistant to antibiotics (*Bacillus clausii*).

### Hemolytic activity

The test for hemolytic activity was performed as per the previously mentioned method [9]. Briefly, bacterial cells were cultured on Columbia blood agar base (Oxoid, Thermo Fisher Scientific, USA), supplemented with 5% (v/v) sheep blood to test their potential to cause distinct forms of hemolysis. Plates were incubated in an aerobic incubator at 37 °C. The observations were made based on the type of hemolysis, and labeled as alpha, beta, and gamma after 24 h and 72 h of incubation periods. A bacterial colony growing on agar is bordered by a greenish discoloration known as alpha hemolysis. Beta hemolysis is the complete breakdown of red blood cells hemoglobin in the presence of the bacterial colony. Gamma hemolysis is indicated by the lack of hemolysis in the area surrounding the bacterial colony. The brownish color of the blood agar plate indicates gamma hemolysis.

### Species identification by 16S rRNA gene sequencing method

One representative isolate from each fingerprinting pattern was selected for PCR amplification of the 16S rRNA gene to identify various bacterial species. The primers 27F (5′-AGAGTTTGATCCTGGCTCAG-3′) and 1492R (5′-TACGGYTACCTTGTTACGACTT-3′) were used to amplify DNA fragments of around 1 kb (equivalent to the size of the 16S rRNA gene). Template DNA, 2 μM primer concentration, and 20 μL Megamix were used to make the reaction mixture (40 μL). An initial denaturation step (94 °C, 5 min), 30 cycles of denaturation (94 °C for 30 s), annealing (58 °C for 30 s), extension (72 °C for 1 min), and a final extension phase (72 °C for 7 min) were performed in a thermal cycler. The PCR products were run on 0.8% (w/v) agarose gels, purified with genomic gel and PCR clean-up and quantified using the gene ruler marker molecular weight standard. The samples were then sent for 16S rRNA gene sequencing analysis as described earlier [22, 29].

The collected rRNA sequences were converted to FastQ format. The FastQ files containing information on the sequences of the studied areas of the 16S rRNA gene, as well as information on the reliability of reading each nucleotide, were generated as a result of the sequencing. The preliminary bioinformatic processing was performed by combining forward and backward reads, filtering sequences with low individual nucleotide readings and chimeric sequences, distributing reads based on barcode sequences, and removing technical sequences using ChromasPro version 2.1.10. The taxonomic verification was done by running the processed sequences using BLASTn [30] against the NT library. The purity of formulation for bacillus was obtained by comparing the % similarity. Phylogenetic analysis (supplementary graphs) was performed using the neighbor-joining method according to the best model identified by MEGA11 version 11.0.11 using the bootstrap test with 1000 replicates [31].

## Results

### Counting of viable bacteria using the plate method

*Bacillus clausii* spores were counted using the plate method. As indicated in Table 3, spores count in BACIPRO® (2.01 × 10^9^), ENTEROGERMINA® (2.10 × 10^9^), and TUFPRO® (2.08 × 10^9^) products matched the label claim of 2 × 10^9^ spores/5 mL. The result indicates a marginal deviation in the colony count of the specified products. However, a lower number of bacterial counts were observed in β-LOCK (2.59 × 10^8^), BENEGUT® (4.50 × 10^8^), PROCILLUS® (2.65 × 10^8^), and PROALANA-B® (5.90 × 10^8^) contrary to the details provided in their label claim. Moreover, as presented in section 3.4, colonies from β-LOCK® and PROCILLUS® products displayed a sequence similarity with *Ralstonia mannitolilytica* and *Paenibacillus dendritiformis*; and it is believed that the bacterial spores count in these 2 products may belong to these species.

**Table 3:** Count of the spore formers in different *Bacillus Clausii* products

SN	Product Name	Batch No. (Spore count)		
1.	BACIPRO® (CFU/5 mL)	B0421: 2.30 × 10^9^	B0621: 2.05 × 10^9^	B0521: 1.71 × 10^9^
2.	ENTEROGERMINA® (CFU/5 mL)	OI197: 2.07 × 10^9^	OI107: 2.04 × 10^9^	OI131: 2.20 × 10^9^
3.	TUFPRO® (CFU/5 mL)	ZEY009: 2.11 × 10^9^	ZEY008: 2.14 × 10^9^	ZEY007: 2.00 × 10^9^
4.	PROALANA-B® (CFU/5 mL)	CBO4620: 5.90 × 10^8^		
5.	PROCILLUS® (CFU/5 mL)	CTO1720: 2.65 × 10^8^		
6.	β-LOCK® (CFU/5 mL)	BC20001: 2.59 × 10^8^		
7.	BENEGUT® (CFU/5 mL)	VBF0134: 4.50 × 10^8^		

### Antibiotic susceptibility profile of the probiotic products

All the 7 *Bacillus clausii* spore suspension products were tested for antibiotic susceptibility by disc-diffusion technique. As presented in Table 4, the zone of inhibition (susceptibility) of test antibiotics ranged from 0 to 39 mm for *Bacillus clausii* strain obtained from BACIPRO®, ENTEROGERMINA®, β-LOCK®, BENEGUT®, PROCILLUS®, PROALANA-B®, and TUFPRO®. Out of the 7 products, BENEGUT® displayed maximum resistance (out of 31 antibiotics 19 showed no zone of inhibition) and β-LOCK® showed maximum susceptibility [out of 31 antibiotics 24 showed a zone of inhibition (range 13–34 mm)]. Moreover, as listed in Table 5, most of the broad-spectrum antibiotics were found to be resistant to the chosen products. All the 7 products were identified to be resistant to cefepime, metronidazole, erythromycin, azithromycin, and nystatin as indicated by the presence of no inhibition zone (Table 4). The results indicate that most of the selected probiotic products are resistant to major antibiotics.

**Table 4:** Inhibition zone diameter (mm) of the test antibiotics

Name of Antibiotic (Disc concentration)	Inhibition zone diameter (mm)						
	BACIPRO®	BENEGUT®	ENTEROGERMINA®	PROCILLUS®	PROALANA-B®	β-LOCK®	TUFPRO®
Clarithromycin (15 mcg)	0	0	0	0	0	25	0
Cefazolin (30 mcg)	12	0	0	13	22	37	12
Cefoperazone (75 mcg)	14	27	10	22	27	23	15
Gentamycin (50 mcg)	26	0	33	30	26	32	24
Cefixime (5 mcg)	0	0	0	0	0	0	0
Chloramphenicol (10 mcg)	0	0	0	11	16	13	0
Clindamycin (10 mcg)	0	0	0	0	22	0	0
Streptomycin (25 mcg)	17	0	14	20	0	20	15
Fluconazole (10 mcg)	0	0	0	0	0	0	0
Metronidazole (5 mcg)	0	0	0	0	0	0	0
Novobiocin (30 mcg)	18	0	0	18	22	14	20
Ciprofloxacin (10 mcg)	28	25	34	32	32	34	30
Erythromycin (5 mcg)	0	0	0	0	0	27	0
Erythromycin (15 mcg)	0	0	0	0	0	0	0
Amoxicillin (25 mcg)	16	10	0	16	0	23	18
Rifampicin (5 mcg)	0	14	0	18	14	17	0
Ofloxacin (5 mcg)	20	15	19	22	29	29	20
Penicillin-G (10 mcg)	27	26	19	28	21	22	25
Azithromycin (15 mcg)	0	0	0	0	0	0	0
Neomycin (30 mcg)	19	0	17	18	0	19	18
Kanamycin (5 mcg)	12	0	16	10	0	14	12
Streptomycin (10 mcg)	13	0	13	16	15	18	12
Cefdinir (5 mcg)	0	0	10	0	0	16	0
Straconazole (10 mcg)	20	18	21	18	28	27	20
Tetracycline (10 mcg)	16	18	10	20	20	25	15
Amoxiclav (30 mcg)	38	39	38	30	32	26	30
Kanamycin (30 mcg)	14	0	18	12	10	14	14
Fusidic acid (30 mcg)	16	14	0	22	22	22	20
Amikacin (30 mcg)	26	12	20	17	20	20	26
Nystatin (75 mcg)	0	0	0	0	0	0	0
Nalidixic acid (30 mcg)	17	13	0	0	19	23	18

**Table 5:** Antibiotic susceptibility test (R: Resistant and S: Susceptible)

**Name of the antibiotic**	BACIPRO^**®**^		BENEGUT^**®**^		ENTEROGERMIN^**®**^		PROCILLUS^**®**^		PROALANA-B^**®**^		β- LOCK^**®**^		TUFPRO^**®**^	
	R	S	R	S	R	S	R	S	R	S	R	S	R	S
Clarithromycin	✔	❌	❌	✔	✔	❌	✔	❌	✔	❌	❌	✔	✔	❌
Cefazolin	❌	✔	❌	✔	✔	❌	❌	✔	❌	✔	❌	✔	❌	✔
Cefoperazone	❌	✔	❌	✔	❌	✔	❌	✔	❌	✔	❌	✔	❌	✔
Cefixime	✔	❌	✔	❌	✔	❌	✔	❌	✔	❌	✔	❌	✔	❌
Chloramphenicol	✔	❌	❌	✔	✔	❌	❌	✔	❌	✔	❌	✔	✔	❌
Streptomycin	❌	✔	❌	✔	❌	✔	✔	❌	✔	❌	❌	✔	❌	✔
Fluconazole	✔	❌	❌	✔	✔	❌	✔	❌	✔	❌	✔	❌	✔	❌
Metronidazole	✔	❌	❌	✔	✔	❌	✔	❌	❌	✔	✔	❌	✔	❌
Novobiocin	❌	✔	❌	✔	✔	❌	❌	✔	❌	✔	❌	✔	❌	✔
Ciprofloxacin	❌	✔	✔	❌	❌	✔	❌	✔	❌	✔	❌	✔	❌	✔
Erythromycin	✔	❌	❌	✔	✔	❌	❌	✔	❌	✔	❌	✔	✔	❌
Erythromycin	✔	❌	❌	✔	✔	❌	✔	❌	✔	❌	✔	❌	✔	❌
Amoxicillin	❌	✔	✔	❌	✔	❌	✔	❌	✔	❌	❌	✔	❌	✔
Rifampicin	✔	❌	✔	❌	✔	❌	❌	✔	❌	✔	❌	✔	✔	❌
Ofloxacin	❌	✔	✔	❌	❌	✔	❌	✔	❌	✔	❌	✔	❌	✔
Penicillin-G	❌	✔	✔	❌	❌	✔	❌	✔	❌	✔	❌	✔	❌	✔
Azithromycin	✔	❌	❌	✔	✔	❌	✔	❌	✔	❌	✔	❌	✔	❌
Neomycin	❌	✔	✔	❌	❌	✔	✔	❌	✔	❌	❌	✔	❌	✔
Kanamycin	❌	✔	❌	✔	❌	✔	✔	❌	❌	✔	❌	✔	❌	✔
Streptomycin	❌	✔	❌	✔	❌	✔	❌	✔	❌	✔	❌	✔	❌	✔
Cefdinir	✔	❌	❌	✔	❌	✔	✔	❌	✔	❌	❌	✔	✔	❌
Itraconazole	❌	✔	✔	❌	❌	✔	✔	❌	❌	✔	❌	✔	❌	✔
Tetracycline	❌	✔	✔	❌	❌	✔	❌	✔	❌	✔	❌	✔	❌	✔
Amoxiclav	❌	✔	✔	❌	❌	✔	❌	✔	❌	✔	❌	✔	❌	✔
Kanamycin	❌	✔	❌	✔	❌	✔	❌	✔	❌	✔	❌	✔	❌	✔
Fusidic acid	❌	✔	✔	❌	✔	❌	❌	✔	❌	✔	❌	✔	❌	✔
Amikacin	❌	✔	✔	❌	❌	✔	❌	✔	✔	❌	❌	✔	❌	✔
Nystatin	✔	❌	✔	❌	✔	❌	✔	❌	✔	❌	✔	❌	✔	❌
Nalidixic acid	✔	❌	✔	❌	✔	❌	❌	✔	✔	❌	❌	✔	✔	❌
Gentamycin	❌	✔	✔	❌	❌	✔	❌	✔	❌	✔	❌	✔	❌	✔
Clindamycin	✔	❌	❌	✔	✔	❌	❌	✔	❌	✔	✔	❌	✔	❌

### Haemolytic activity

**Fig. 3:**
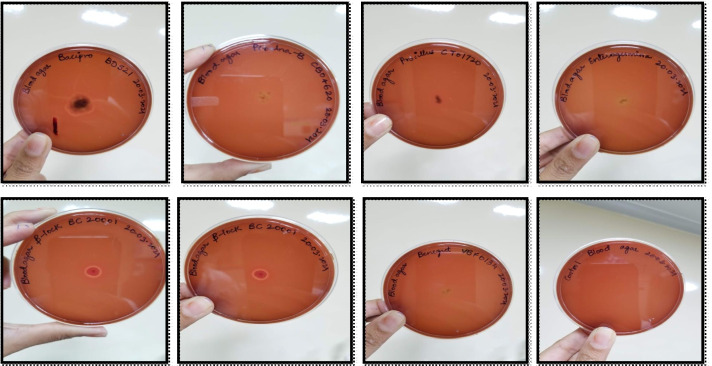
The hemolytic activity test using *Bacillus clausii* probiotic products

As depicted in Fig. 3, 6 out of 7 products showed no degradation in the agar plate, thereby indicating gamma-hemolysis activity. However, β-LOCK® probiotic exhibited beta-hemolysis as represented by the complete breakdown of hemoglobin in the proximity of a bacterial colony.

### Species identification using 16S rRNA gene sequence analysis

The 16S rRNA gene sequencing results are tabulated in Table 6. The bacterial colonies from BACIPRO®, ENTEROGERMINA®, BENEGUT®, PROALANA-B®, and TUFPRO® displayed sequence identity of *Bacillus clausii* species, according to sequence analysis of the 16S rRNA gene amplicon collected from the isolates (Table 6). However, colonies sequenced from β-LOCK® and PROCILLUS® products showed high sequence similarity with *Ralstonia mannitolilytica* and *Paenibacillus dendritiformis*.

**Table 6:** 16S rRNA gene sequencing data of seven probiotic products

**SN**	**Product Name**	**Organism**	**Number of Hits**	**Per. Ident**	**Accession Number**
1.	BACIPRO^**®**^	*Alkalihalobacillus clausii*	113	96.95%	MH114929.1
		*Alkalihalobacillus rhizosphaerae*	4	96.95%	KT595230.1
		*Bacillus* sp. B36	1	96.95%	KC492106.1
		*Bacillus* sp. NBRC 101259	1	96.95%	AB681430.1
		*Bacillus* sp. BAB-3415	1	96.88%	KF917146.1
2.	ENTEROGERMINA^**®**^	*Alkalihalobacillus clausii*	95	97.69%	MK859951.1
		*Alkalihalobacillus rhizosphaerae*	6	97.68%	MT981109.1
		*Bacillus clausii* KSM-K16	1	97.46%	KR350629.1
		*Bacillus* sp. NBRC 101257	1	97.46%	AB681428.1
3.	BENEGUT^®^	*Alkalihalobacillus clausii*	114	97.34%	EU977787.1
		*Alkalihalobacillus rhizosphaerae*	6	97.19%	KT595230.1
		*Bacillus* sp. BAB-3415	1	97.11%	KF917146.1
		*Bacillus clausii* KSM-K16	1	97.19%	KR350629.1
		*Bacillus* sp. NBRC 101259	1	97.19%	AB681430.1
4.	β-LOCK^®^	*Ralstonia mannitolilytica*	13	97.82%	LN890110.1
		*Ralstonia pickettii*	23	97.82%	MK934372.1
		*Uncultured Ralstonia*sp.	69	97.82%	KX405177.1
5.	PROCILLUS^**®**^	*Paenibacillus popilliae*	18	96.80%	KC107788.1
		*Paenibacillus thiaminolyticus*	19	96.72%	LC379101.1
		*Paenibacillus dendritiformis*	48	96.72%	MH555122.1
6.	PROALANA-B^**®**^	*Alkalihalobacillus clausii*	114	96.54%	EU977787.1
		*Alkalihalobacillus* rhizosphaerae	5	96.32%	MT903021.1
		*Bacillus* sp. NCBR 101258	1	96.27%	AB681429.1
		*Bacillus* sp. NBRC 101259	1	96.27%	AB681430.1
		*Bacillus clausii* KSM-K16	1	96.27%	KR350629.1
7.	TUFPRO^®^	*Alkalihalobacillus clausii*	83	91.61%	KT719550.1
		*Alkalihalobacillus rhizosphaerae*	12	91.38%	MK386746.1
		*Bacillus sp. NBRC 101258*	1	91.15%	KF917146.1
		*Bacillus sp. BAB-3415*	1	91.15%	KF917146.1
		*Bacillus sp. mixed culture J4-45*	1	91.25%	KR029228.1

## Discussion

Our study corroborated the label count (2 × 10^9^ CFU/5 mL) in BACIPRO®, ENTEROGERMINA®, and TUFPRO® products containing *Bacillus clausii*. The *Bacillus clausii* probiotics are known to impart a variety of health benefits such as recovery from inflammatory bowel illness and acute diarrhoea in children under the age of 5 years as well as in adults [32]. The safety and tolerability of *Bacillus clausii* probiotics have been well studied. Treatment with *Bacillus clausii* (2 × 10^9^ CFU/5 mL) for 10 days was found to be effective against acute diarrhoea and safe in humans [7]. Several other clinical studies have also validated the safety profile of other *Bacillus clausii* probiotics [15, 33]. As the efficacy of probiotics is associated with the strain-specific phenotype and the number of live bacteria, we performed both qualitative and quantitative tests for some marketed *Bacillus clausii* products. In the quantitative assays, the label information about the number of *Bacillus clausii* count was found to match the label claims for BACIPRO®, ENTEROGERMINA®, and TUFPRO® formulations. However, we noted some mismatch in the claimed bacterial counts in β-LOCK®, BENEGUT®, PROCILLUS®, and PROALANA-B® products. While analysis of these samples was done in triplicate, a lower number of vegetative cell counts in the latter 4 products may be limited to the specific batch. A similar type of mismatch concerning the number of *Bacillus clausii* counts has been reported in the commercially available probiotic products marketed in Italy [34]. The study also suggested the importance of independent laboratory analysis in the quality check of label indications. Since the count of the viable bacteria is one of the critical elements in defining probiotic effectiveness, deviation from the actual content of strains in probiotic formulations should be taken into consideration. We propose that stringent quality control measures may provide better clinical benefits to the patients.

Endospore-forming *Bacillus clausii* are aerobic, gram-positive bacteria, and resistant to broad-spectrum antibiotics [28]. Thus, we evaluated the antibiotic susceptibility of *Bacillus clausii* suspension against 31 antibiotics using the disc-diffusion technique in the selected 7 probiotics. Antibiotic susceptibility results indicated resistance of BACIPRO®, ENTEROGERMINA®, β-LOCK®, BENEGUT®, PROCILLUS®, PROALANA-B®, and TUFPRO® to cefepime, metronidazole, erythromycin, azithromycin, and nystatin. Earlier studies also reported antibiotic resistance of *Bacillus clausii* strains in ENTEROGERMINA® [35]. The antibiotic-resistant properties of probiotics help in restoring commensal microflora and survival during concomitant treatment with antibiotics [36]. The analysis of whole-genome sequencing for antibiotic-resistant and transferable genes of each *Bacillus clausii* strain used in their probiotic products is not available, except few products [9, 37]. It has been reported that the production of an aminoglycoside inactivating enzyme by the aadD2 chromosomal gene in *Bacillus clausii* confers resistance to aminoglycosides [33] and a chromosomal mutation may be causal to the resistance of *Bacillus clausii* to rifampicin [33, 37]. Moreover, the expression of the CAT by the catBcl gene in probiotics containing *Bacillus clausii* has been reported to result in resistance to chloramphenicol [36]. *Bacillus clausii* containing probiotics contain several classes of beta-lactamases that are resistant to penicillin such as ampicillin [38]. Taken together, most of the commercially available *Bacillus clausii* probiotics are antibiotic-resistant, and concomitant consumption with antibiotics may not affect the viability of the *Bacillus clausii* containing probiotics.

The evaluation of hemolytic activity was carried out as required by the European Food Safety Authority (EFSA). Out of the 7 probiotic products, only one β-LOCK® probiotic formulation showed the sign of β-hemolysis. This result indicates the requirement for stringent regular quality checks of commercially available probiotic products. All the 7 probiotics were also subjected to the species level investigation using 16S rRNA gene sequencing. The data confirmed the genera of *Bacillus* and *Bacillus clausii* species in BACIPRO®, ENTEROGERMINA®, PROALANA-B®, BENEGUT®, and TUFPRO® products. However, the presence of *Ralstonia mannitolilytica*/*pickettii* and *Paenibacillus dendritiformis/popilliae* species were noted in β-LOCK**®** and PROCILLUS®, respectively. As 16S rRNA gene sequencing data has been considered an integral asset for distinguishing proof and phylogenetic investigation of microscopic organisms, the detection of other strains in commercial probiotics may raise clinical concerns. However, limitation of this study is that the bacterial genus level differences were not identified by this gene sequencing method, as shotgun metagenomics analysis was not done. We noted a high consistency in the label information of BACIPRO®, ENTEROGERMINA®, PROALANA-B®, BENEGUT®, and TUFPRO® products, which exhibited match with superior efficacy as well as popularity.

In conclusion, the number of viable bacterial counts did not match with the specified data in 4 four probiotic products, and 2 out of 7 probiotic formulations differed qualitatively concerning the label information. It is proposed that a regular and rigorous quality control process should be adopted to ensure the asserted label information in the probiotics. We suggest that a periodic reconnaissance is essential to control the clinical effectiveness of commercially available probiotic products.

### Data availability

The datasets generated and/or analyzed during the current study are available in the INSDC repository, and the accession number for the raw data generated with the 16S rRNA gene sequencing reported in this paper is BioProject PRJDB13145.

## Supplementary Information

**Additional file 1.**

**Additional file 2.**

**Additional file 3.**

**Additional file 4.**

**Additional file 5.**

**Additional file 6.**

**Additional file 7.**

## Data Availability

16S rRNA data is deposited in the INSDC repository having an accession number: BioProject PRJDB13145.
