# Supplementary figures and images for: Quantitative and qualitative characterization of commercially available oral suspension of probiotic products containing Bacillus Clausii spores
Source: BMC Microbiol. 2022 Sep 17;22:217. doi: 10.1186/s12866-022-02631-w (PMC9482283; doi:10.1186/s12866-022-02631-w)

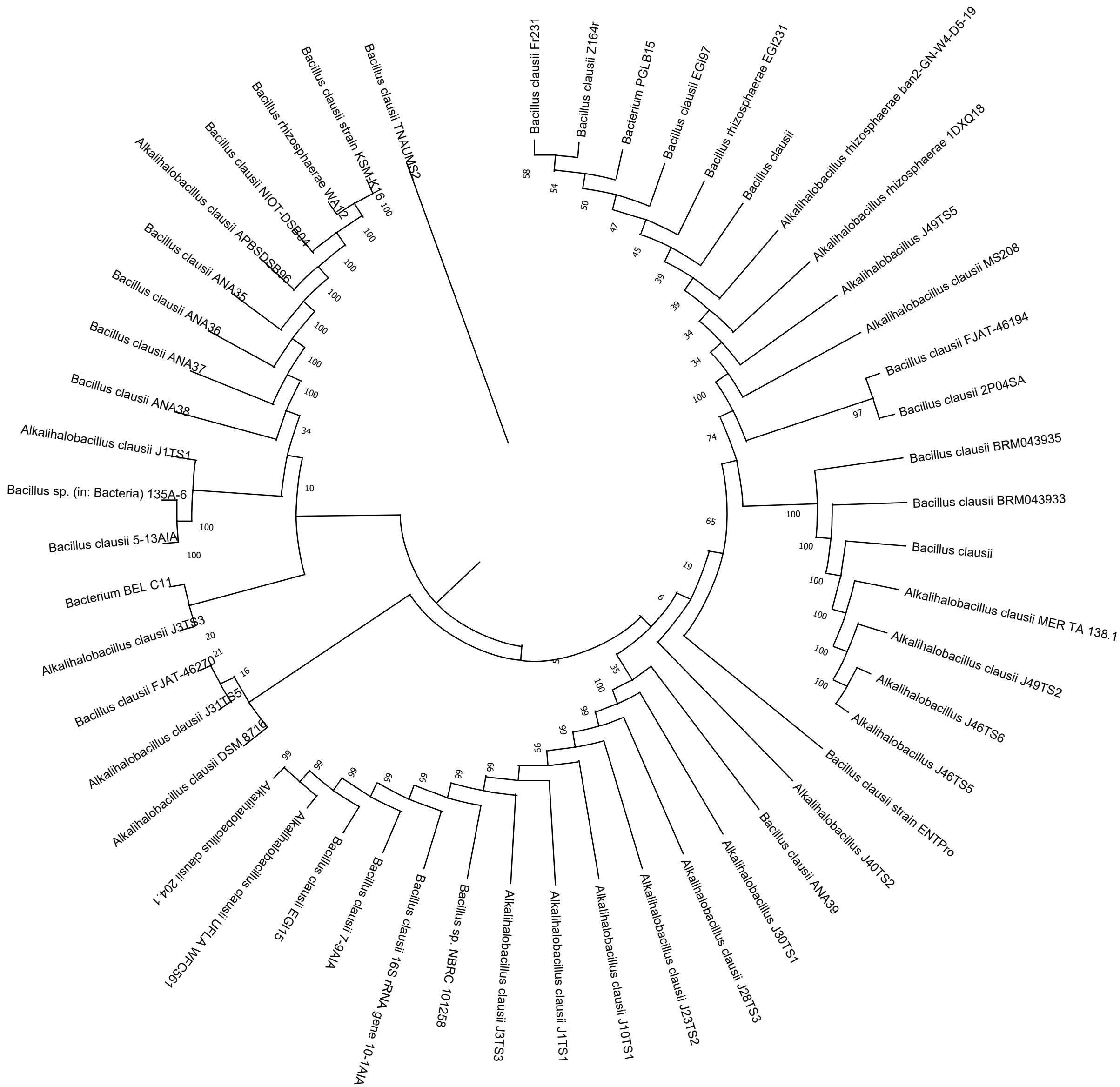

Supplement: Supplementary file 1 — Additional file 1. [file 12866_2022_2631_MOESM1_ESM.pdf]

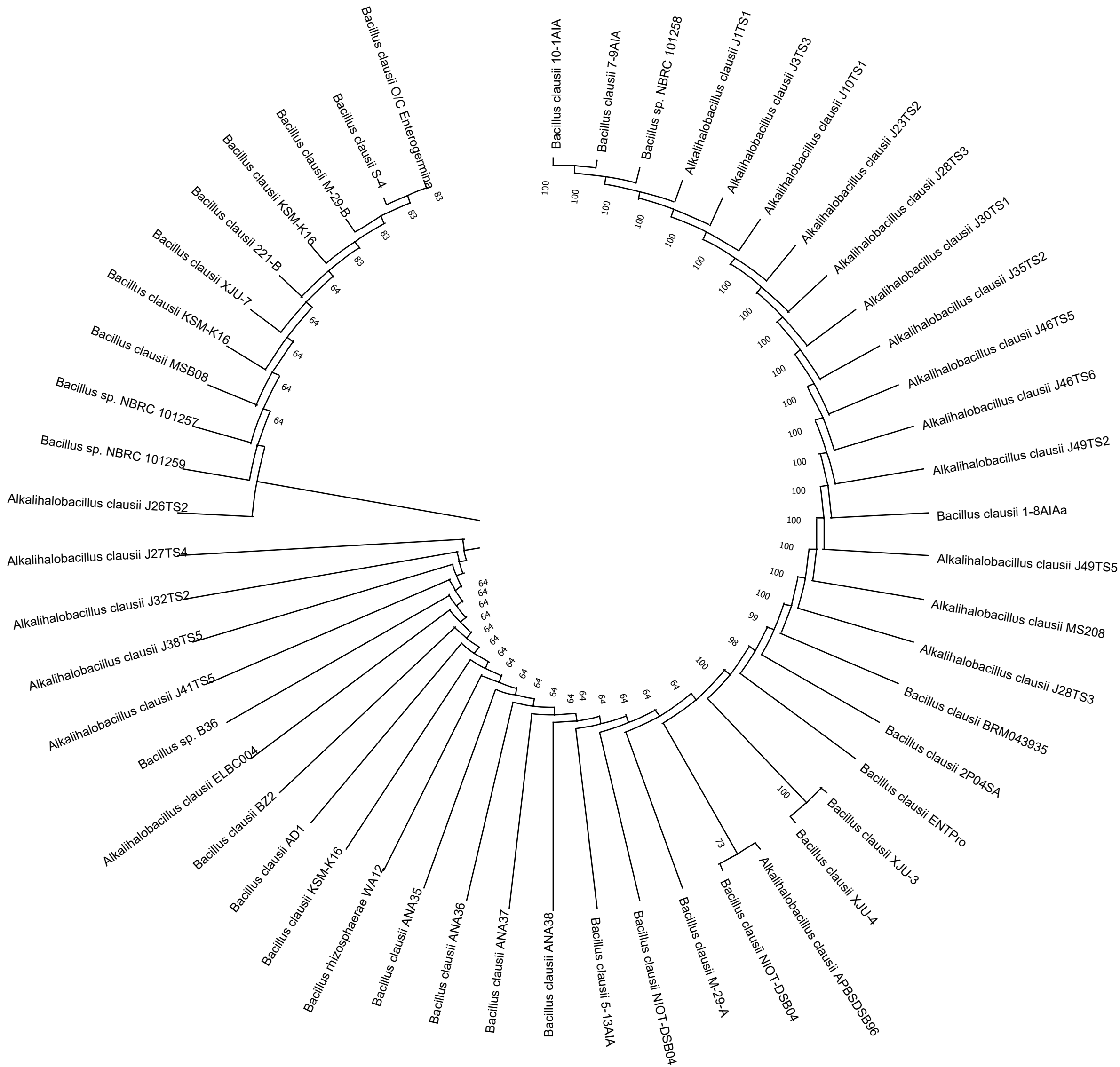

Supplement: Supplementary file 2 — Additional file 2. [file 12866_2022_2631_MOESM2_ESM.pdf]

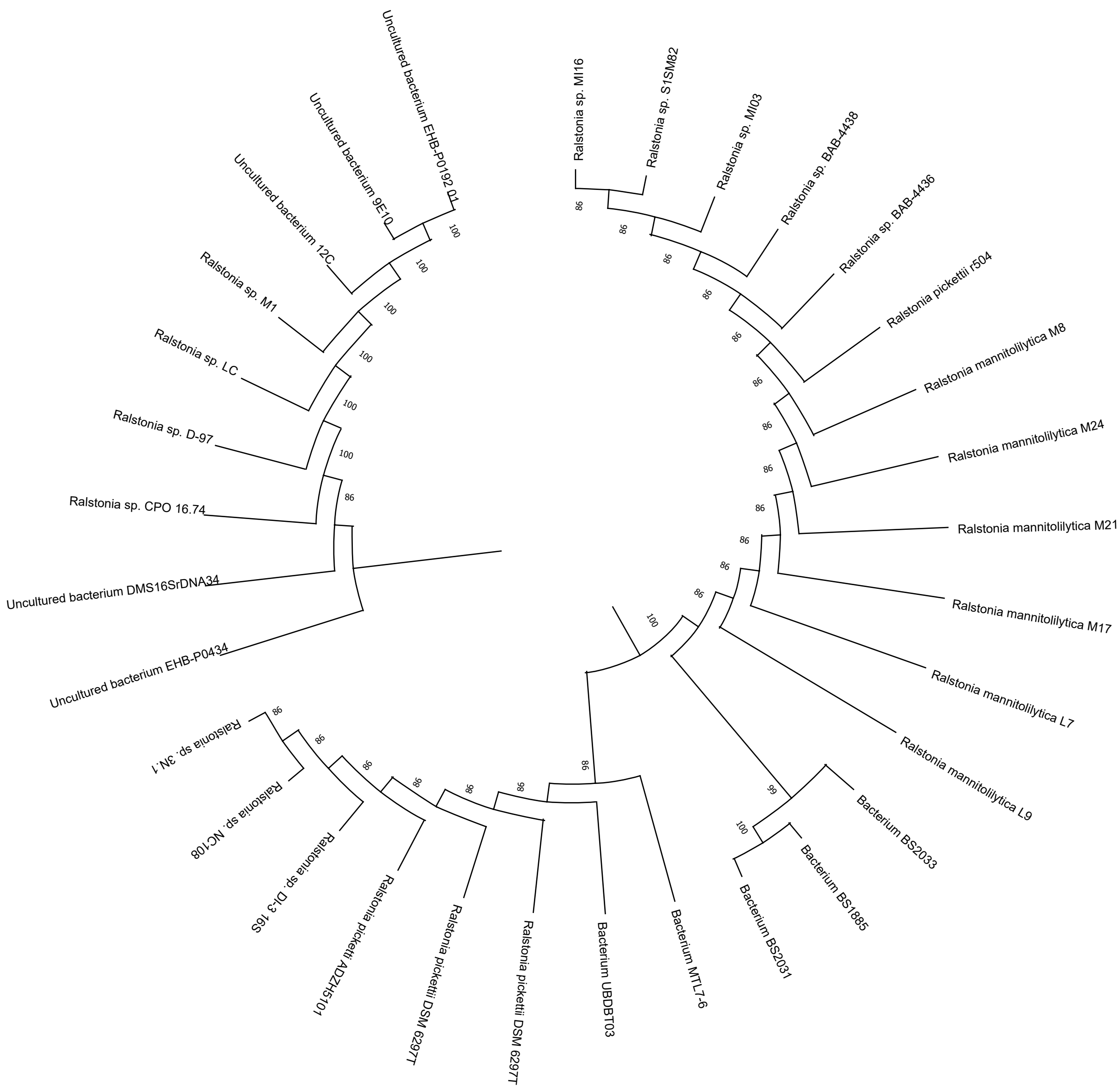

Supplement: Supplementary file 3 — Additional file 3. [file 12866_2022_2631_MOESM3_ESM.pdf]

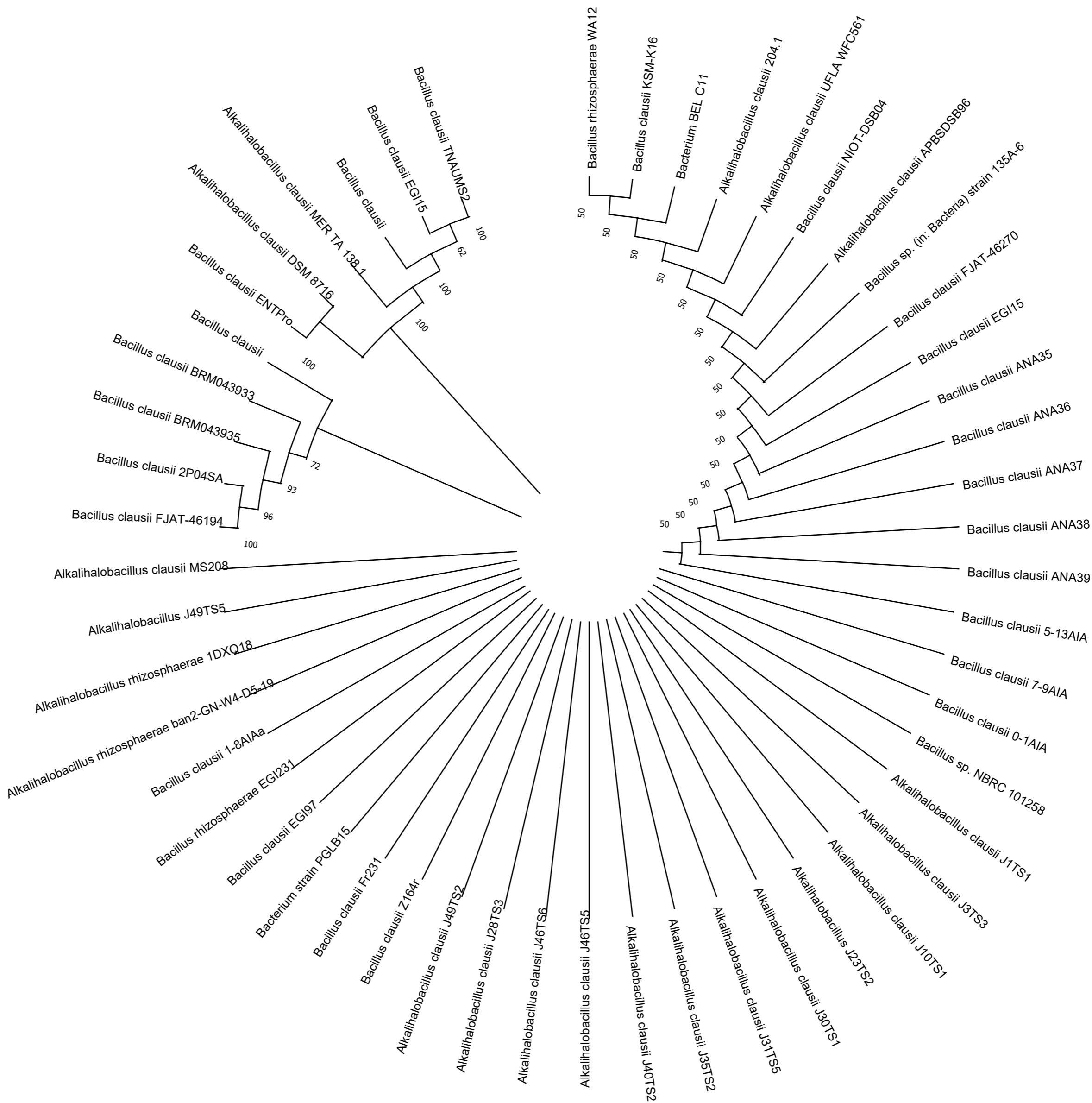

Supplement: Supplementary file 4 — Additional file 4. [file 12866_2022_2631_MOESM4_ESM.pdf]

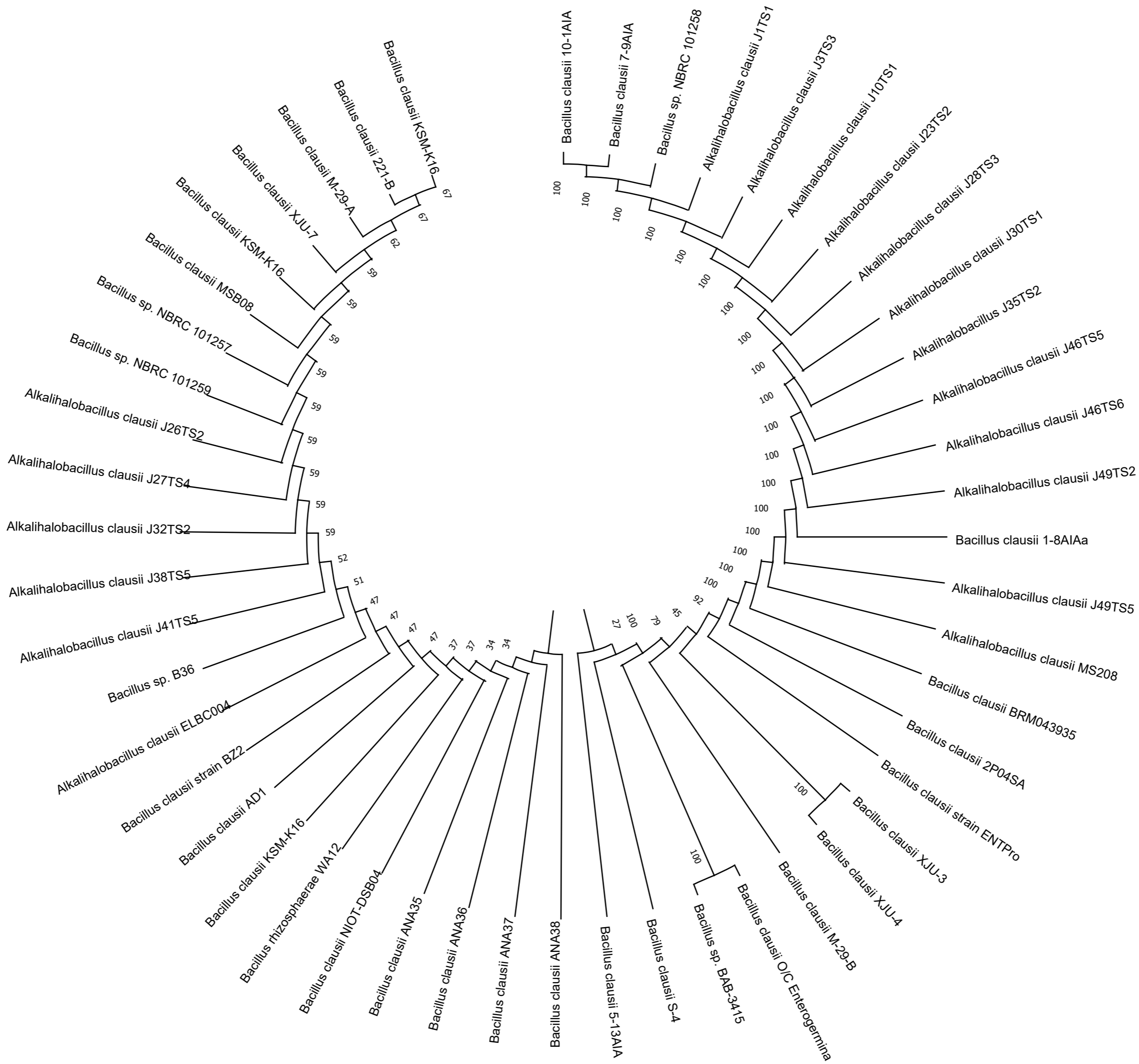

Supplement: Supplementary file 5 — Additional file 5. [file 12866_2022_2631_MOESM5_ESM.pdf]

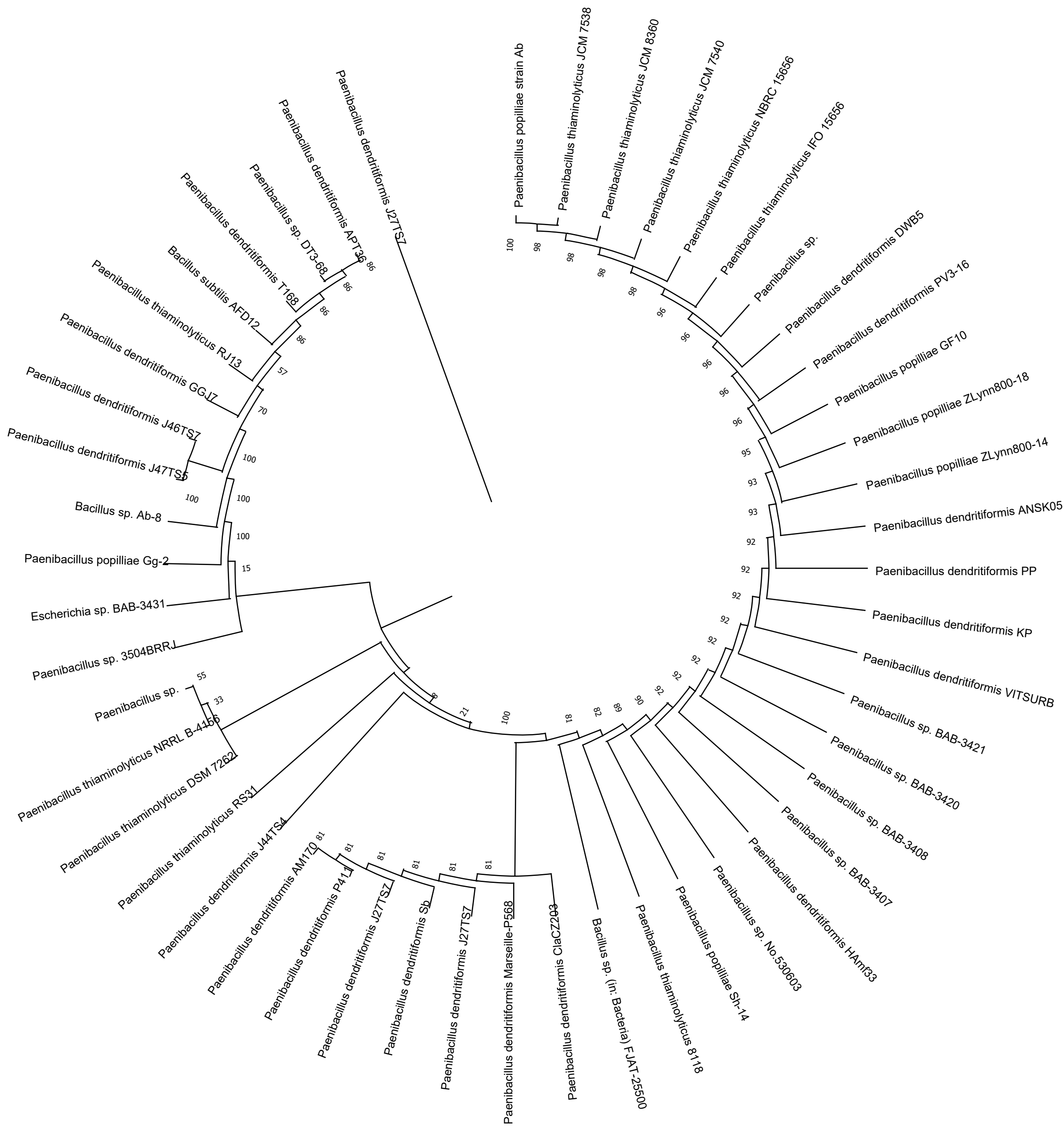

Supplement: Supplementary file 6 — Additional file 6. [file 12866_2022_2631_MOESM6_ESM.pdf]

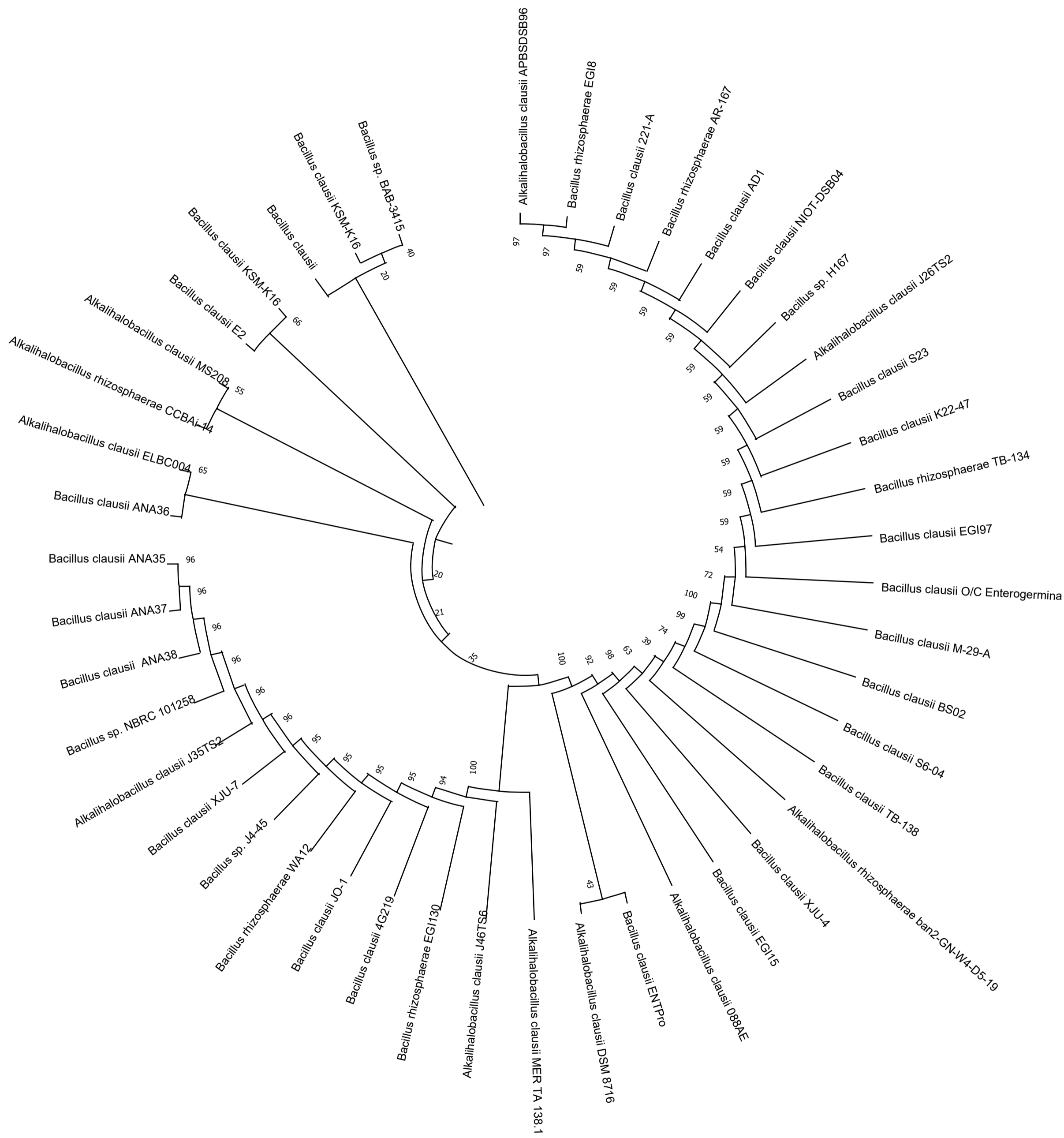

Supplement: Supplementary file 7 — Additional file 7. [file 12866_2022_2631_MOESM7_ESM.pdf]
